# Radioresistant but Alectinib‐Responsive Isolated Intramedullary ALK‐Positive Histiocytosis

**DOI:** 10.1155/crh/1712521

**Published:** 2026-02-01

**Authors:** Joshua Van Allen, David Raggay, Brendan Killory, Joseph DiGiuseppe, Mark Dailey

**Affiliations:** ^1^ Department of Hematology and Oncology, University of Connecticut School of Medicine, Farmington, Connecticut, USA, uchc.edu; ^2^ Department of Pathology, Hartford Hospital, Hartford, Connecticut, USA, hartford.edu; ^3^ Department of Neurosurgery, University of Connecticut School of Medicine, Farmington, Connecticut, USA, uchc.edu; ^4^ Department of Neurosurgery, Hartford Hospital, Hartford, Connecticut, USA, hartford.edu; ^5^ Deparment of Hematology and Oncology, Hartford Hospital, Hartford, Connecticut, USA, hartford.edu

**Keywords:** alectinib, ALK, histiocytosis

## Abstract

A 56‐year‐old woman with a history of C4–C5 myelomeningocele repair as a newborn and cervical syringomyelia presented with one week of rapidly worsening bilateral lower extremity weakness and numbness, saddle anesthesia, and bladder incontinence. MRI of the entire spine revealed a 1.5 × 0.5 cm homogenously enhancing intramedullary lesion at T7–T8 with associated cord edema and rostral syrinx formation. MRI of the brain and FDG PET–CT scan were unremarkable. She was taken to the operating room for a T6–T8 laminectomy and biopsy. H&E staining revealed a relatively dense mononuclear‐cell infiltrate, which comprised numerous medium‐sized cells with round, irregular, or reniform nuclei, slightly dispersed chromatin, and relatively abundant eosinophilic, and occasionally, somewhat vacuolated cytoplasm. Immunohistochemical staining was strongly positive for CD163, PU.1, CD68, and ALK. FISH studies demonstrated ALK rearrangement in 70% of nuclei. Next‐generation sequencing including both DNA and RNA testing on a formalin‐fixed, paraffin‐embedded tissue sample detected a KIF5B/ALK gene fusion. She received radiotherapy with 2000 cGY in 200 cGy fractions to T6–T9 with no change in lesional size or enhancement. She was started on the ALK inhibitor alectinib. Subsequent MRI showed a complete response. She has had no evidence of disease recurrence on alectinib for 18 months. ALK‐positive histiocytosis is a recently described distinct clinicopathologic entity. Our case is notable for older age at diagnosis, isolated intramedullary involvement, and radioresistance but later marked targeted‐therapy response, thus furthering the understanding of the spectrum of ALK‐positive histiocytosis biology.

## 1. Introduction

Histiocytoses are neoplastic disorders characterized by histiocytic infiltration of virtually any tissue or organ of the body, with the majority harboring mutations in BRAF or MAP2K1 genes and less frequently involving alterations in CSF1R, ALK, RET, NTRK, and other kinase genes [1, 2]. ALK‐positive histiocytosis, in particular, is a rare neoplasm and a relatively recently described distinct clinicopathologic entity that was first described in young infants by Kemps et al. in 2008 [3–5]. This entity is typically seen in younger individuals, and central nervous system (CNS) involvement is a common manifestation. However, few cases present with isolated CNS disease [4]. This entity is frequently characterized by the presence of the KIF5B–ALK gene fusion [5]. While ALK rearrangements are well established in non–small‐cell lung cancer (NSCLC), they are extremely uncommon in other cancer types [6].

## 2. Case Presentation

We present the case of a 56‐year‐old woman with a history of C4–C5 myelomeningocele repair as a newborn and cervical syringomyelia who presented with one week of rapidly worsening bilateral lower extremity weakness and numbness, saddle anesthesia, and bladder incontinence. Her neurological examination was remarkable for 4 out of 5 bilateral hip flexor strength, 3+ bilateral patella and Achilles reflexes, bilateral ankle clonus, and a broad‐based, spastic gait. At baseline, she had bilateral upper extremity numbness and intermittent shooting pain from a prior C4–C5 myelomeningocele repair with persistent spinal cord tethering.

**Figure 1:**
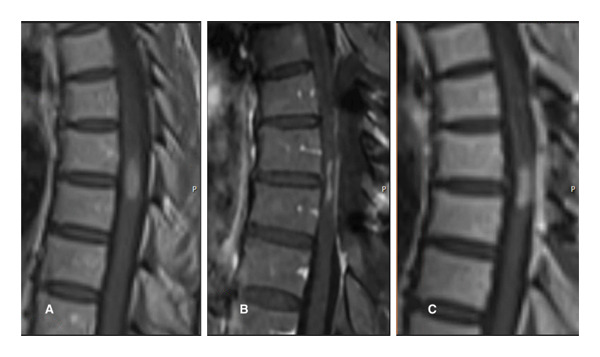
Sagittal T1 postcontrast MRI revealing homogenously enhancing intramedullary lesion at T7–T8 with associated cord edema and rostral syrinx formation immediately before (A) and after (B) T6–T8 laminectomy/laminoplasty for biopsy/subtotal resection. Subsequent MRI at 1‐month postop (C) reveals regrowth of the lesion.

She was admitted to the hospital and started on oral dexamethasone. She reported modest improvement in her symptoms. MRI of the entire spine revealed a 1.5 × 0.5 cm homogenously enhancing intramedullary lesion at T7–T8 with associated cord edema and rostral syrinx formation (Figure 1(A)). MRI of the brain and 18‐fluorine‐FDG CT–PET scan of the whole body were both unremarkable. Findings were suggestive of a neoplasm, and she was taken to the operation room for a T6–T8 laminectomy and biopsy. Intraoperatively, the lesion was greyish tan, appeared grossly consistent with a neoplasm, and had a well‐defined tissue plane with the surrounding spinal cord. The resection was aborted when pathology was suggestive of an inflammatory process, and there was a significant (greater than 50% decrease in amplitude) transient reduction in motor evoked potentials. Postoperatively, her strength and sensation were improved from a presurgical assessment.

H&E sections from the spinal cord tumor biopsies revealed a relatively dense mononuclear‐cell infiltrate, which comprised numerous medium‐sized cells with round, irregular, or reniform nuclei, slightly dispersed chromatin, and relatively abundant eosinophilic, and occasionally, somewhat vacuolated cytoplasm (Figure 2).

Figure 2Direct smear (a) and H&E section (b) revealing a relatively dense mononuclear‐cell infiltrate, which comprises numerous medium‐sized cells with round, irregular, or reniform nuclei, slightly dispersed chromatin, and relatively abundant eosinophilic, and occasionally, somewhat vacuolated cytoplasm.

**Figure:**
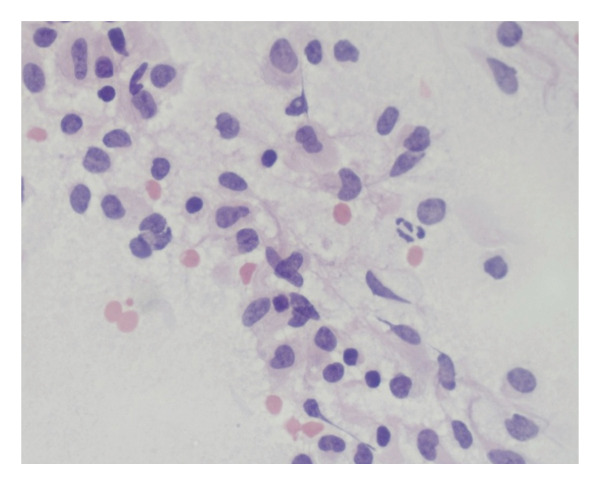
(a)

**Figure:**
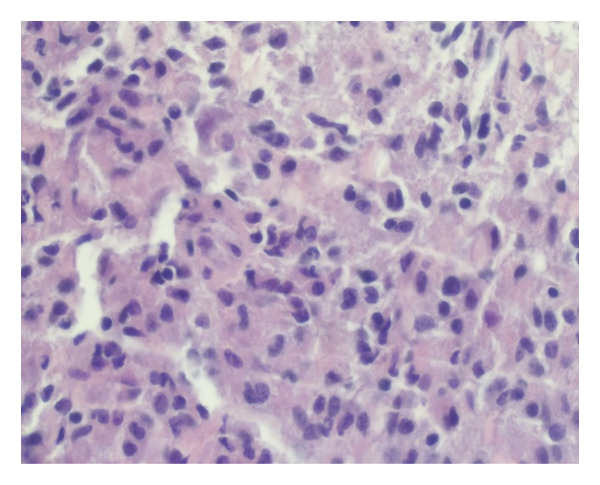
(b)

Comparatively few small lymphocytes with round nuclei and condensed chromatin were admixed, as well as rare large cells with vesicular chromatin. By immunohistochemistry, the tumor cells were strongly positive for the histiocytic marker, CD163, with punctate cytoplasmic staining for two different epitopes of the histiocytic antigen, CD68 (KP1 and PGM1), weak cytoplasmic expression of CD4, and extensive cytoplasmic (but not nuclear) staining for ALK (CD246 and D5F3). The cells also displayed strong nuclear and weak cytoplasmic expression of cyclin D1, with somewhat variable positivity for S100. Most of the cells in the infiltrate were positive for PU.1, and approximately 10%–20% of the cells were Ki‐67+. A summary of the immunohistochemical profile is shown in Table 1.

**Table 1:** Immunohistochemical profile of tumor cells.

Immunohistochemical marker	Result
ALK	+
CD4	+ (weak)
CD68	+
CD163	+
Cyclin D1	+
Ki‐67	+ (10%–20%)
PU.1	+
S100	+ (variable)
CD1a	−
CD21	−
CD23	−
CD30	−
CD123	−
CD138	−
CMV	−
EMA	−
GFAP	−
HSV1/2	−
IDH‐1	−
Neurofilaments	−
OCT2	−
SV40	−
Synaptophysin	−

FISH studies employing a break‐apart probe for ALK demonstrated ALK rearrangement in 70% of nuclei. Next‐generation sequencing including both DNA and RNA testing on a formalin‐fixed, paraffin‐embedded tissue sample from the spinal cord tumor block revealed the KIF5B/ALK in‐frame, activating gene fusion.

**Figure 3:**
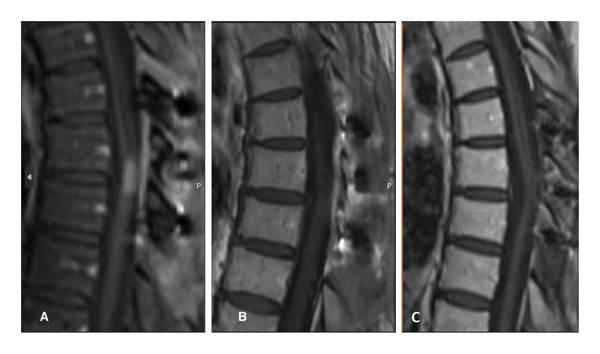
Sagittal T1 postcontrast MRI 1 month after radiation therapy (A) reveals persistent intramedullary enhancement that demonstrates a significant treatment response 2 months (B) and 18 months (C) after initiating alectinib therapy.

Postsurgery MRI revealed regrowth of the lesion (Figure 1(A)). She was also experiencing worsening neurologic symptoms. She then received radiotherapy to T6–T9 with 2000 cGY in 200 cGY fractions via a three‐field technique utilizing 10‐mV photons. After radiotherapy, she had no change in the size of the lesion or enhancement on MRI (Figure 3(A)). She was started on the ALK inhibitor, alectinib. Subsequent MRI showed a complete response. She had no evidence of disease recurrence or progression since starting alectinib 18 months prior (Figures 3(C)). She has continued to show a response to alectinib almost 3 years later.

## 3. Discussion

A review conducted by Kemps et al. in 2022 evaluated 39 patients, including 37 cases with confirmed ALK rearrangements. Of these cases, 31 included pediatric patients with most of these cases involving children aged 3 years or younger. There also appears to be a female predilection in this disease entity. In the Kemps et al. review, the oldest patient with the KIF5B–ALK gene fusion was diagnosed at 41 years of age [3]. More recently, Liu et al. described a case of a 51‐year‐old woman with a lesion infiltrating the brain parenchyma [4].

Alectinib, an ALK inhibitor, is FDA‐approved in ALK‐positive advanced‐stage NSCLC, but its use in ALK‐positive histiocytosis is not well established given the rarity of this condition. However, there have been several reports of the use of ALK inhibitors, such as alectinib, in ALK‐positive histiocytosis, with a reported good response to treatment.

Upon literature review, it appears that our case involves the oldest recorded patient with ALK‐positive histiocytosis harboring the KIF5B–ALK gene fusion. Our case is notable not only for older age at diagnosis but also for isolated intramedullary involvement and radioresistance with later significant response to ALK‐targeted therapy, thus furthering the understanding of the spectrum of ALK‐positive histiocytosis biology.

## Funding

No funding was received for this manuscript.

## Disclosure

This manuscript was also presented at the Northern New England Clinical Oncology Society 2023 Annual Meeting in Bretton Woods, NH, on October 27–28, 2023, and was presented at the Leukemia Lymphoma Society Visionaries 2025 Precision Oncology Educational Symposium in Hartford, CT, on April 5, 2025 https://nnecos.org/abstracts-23.

## Conflicts of Interest

The authors declare no conflicts of interest.

## Data Availability

The data that support the findings of this study are available from the corresponding author upon reasonable request.
